# Testing an online screening for autism in the COVID-19 pandemic: a psychometric study of the Q-CHAT-24 in Chilean toddlers

**DOI:** 10.3389/fpsyt.2024.1363976

**Published:** 2024-06-17

**Authors:** Gabriel Gatica-Bahamonde, Alejandra Mendez-Fadol, Francisca Sánchez-Sepúlveda, Constanza Peñailillo-Diaz, Robin van Kessel, Katarzyna Czabanowska, Andres Roman-Urrestarazu

**Affiliations:** ^1^ Department of International Health, Care and Public Health Research Institute (CAPHRI), Faculty of Health, Medicine and Life Sciences, Maastricht University, Maastricht, Netherlands; ^2^ Sección de Psiquiatría del Niño y del Adolescente, División de Neurociencias, Facultad de Medicina, Pontificia Universidad Católica de Chile, Santiago, Chile; ^3^ Mental Health, Policy and Economics Group, Department of Psychiatry, University of Cambridge, Cambridge, United Kingdom; ^4^ Tremün Lab, Corporación Tremün, Villarrica, Chile; ^5^ Departamento de Pediatría, Facultad de Medicina, Universidad de La Frontera, Temuco, Chile; ^6^ LSE Health, Department of Health Policy, London School of Economics and Political Science, London, United Kingdom; ^7^ Institute of Public Health, University of Cambridge, Cambridge, United Kingdom

**Keywords:** autism spectrum disorder, early detection, online screening, screening, COVID-19, Q-CHAT, autism, telehealth

## Abstract

**Background:**

The aim of this study was to examine some psychometric characteristics of the Chilean-adapted version of the Quantitative Checklist for Autism in Toddlers (Q-CHAT-24) (24) in a group of unselected children (community sample). This version was administered remotely through an online version during the pandemic period to caregivers of children, aged 18–24 months, registered in four primary care polyclinics of the Health Service Araucanía Sur, Chile.

**Methods:**

An intentional non-probabilistic sampling was used. Three hundred and thirteen toddlers were examined. Participants completed an online version of the Q-CHAT-24 which was disseminated through the REDCap platform. Evidence of reliability through internal consistency and evidence of predictive validity through ROC curve analysis were realized.

**Results:**

The mean age of the children evaluated was 21.16 months. The Shapiro-Wilk test revealed that Q-CHAT-24 scores was normally distributed. 71 cases (23.12%) scored 38 points or more on the Q-CHAT-24, qualifying as Autistic Risk. 48 cases (15.63%) were confirmed as autistic through the ADOS-2 Module T. All items were positively correlated with Q-CHAT-24 total score. All items were positively correlated with Q-CHAT-24 total score. Internal consistency was acceptable for the Q-CHAT-24 (Cronbach ´s α=0.78). The internal consistencies were analyzed for the Q-CHAT-24 Factors, and they were good for factor 1 “Communication and Social Interaction” (Cronbach ´s α=0.85) and acceptable for factor 2 “Restrictive and Repetitive Patterns” (Cronbach ´s α=0.74). Receiver operating characteristic (ROC) curve analyses were performed. The AUC values were 0.93 with statistical significance (p<0.01). For the cut-off point of 38, the Sensitivity, Specificity and Youden index values were 0.89, 0.8 and 0.7, respectively. The Positive Predictive Value (PPV) was 86% and the Negative Predictive Value (NPV) was 85%.

**Conclusions:**

In accordance with the objectives of this study, evidence of reliability and predictive validity was demonstrated for the Q-CHAT-24 in this Chilean population. More importantly, this study provides Sensitivity and Specificity data for a remote application version of an autism screening tool already validated in Chile. The implications of this have to do with the possibility of establishing a remote assessment system for children at risk of autism on a population scale.

## 1 Introduction

Autism Spectrum Disorders (ASD, hereafter ‘autism’) are a group of neurodevelopmental conditions characterized by persistent difficulties in communication and social interaction, and the presence of restricted and repetitive patterns of behavior, interests, and activities (1). Autism is of increasing epidemiological importance: recent studies show prevalence between 1.76% (UK) (2) and 2.77% (US) (3).

Early detection of signs of autism allows early referral to intervention programs (4). This is important because there is evidence that early management improves the prognosis and quality of life of children and their families (5, 6), that interventions are more effective at earlier ages (7) and that early intervention results in lower costs for the health care system (8).

Evidence shows that signs of autism can be detected early during the first and second years of life through caregiver’s self-report by screening tool (9). An autism screening tool is a brief assessment that is administered to detect those who exhibit traits of autism and are therefore at risk for the condition (10) and who can thus be referred to early intervention programs within a critical developmental period priced at 36 months of age (6). Evidence shows that early detection through screening reduces the time between diagnosis and initiation of intervention by up to 70% (11). Despite the above, a very small percentage of parents are alerted to their child’s developmental problems through the application of an autism-specific screening test (12) and a percentage between 30% and 50% of children with autism continue to be diagnosed after the age of 6 years (13).

With restrictions on face-to-face and in-person care, the COVID 19 pandemic posed a major challenge to the goal of early detection and diagnosis of autism, requiring rapid adaptation of child care services to the remote mode of care (14, 15). Brunt et al. (2023) (15) found that while all child populations had their assessment and diagnostic processes disrupted in the pandemic, there were glaring disparities for children with autism.

While prior to the pandemic there were studies showing the feasibility and preliminary accuracy of telemedicine early diagnosis methods (16), evidence for remote assessment methods is still limited. Corona et al. (2023) (17) evaluated the diagnosis concordance of autism between a face-to-face assessment and a remote assessment using ELE-ASD-PEDS (TAP) and the Screening Tool for Autism in Toddlers (STAT), in a sample of 144 children aged 17–36 months. The concordance between the face-to-face and remote assessments was 92%. Some of the factors that explained the diagnostic errors in the remote method were the younger age of the children and the better performance in the developmental assessment. Gibbs et al. (2021) (18) conducted a study investigating the acceptability of remote diagnostic procedures in the COVID-19 crisis. In general, caregivers and parents of children with autism felt welcomed in remote settings and their expectations of assessment were met, but the authors emphasized the need for a high structured assessment process. Colombo et al. (2022) (19) published a descriptive and preliminary analysis of a web platform (Web Italian Network for Autism Spectrum Disorder WIN4ASD) that used CHAT as a tool for early autism screening in a limited care setting.

Chile is a South American country with a population of approximately 19 million inhabitants (20), which despite being considered a high-income country, lives with significant health disparities (21). To address inequalities in the diagnosis and detection of autism, Chile has legislated a new autism law in 2023 that establishes a regulatory framework for the comprehensive care and protection of people with this condition, with the aim of improving their quality of life and promoting their social inclusion (22). The law makes explicit the centrality of early detection in the approach to the condition. A recent systematic review evaluated the tools validated in Chile for early detection of autism, finding only three screening tools (23). Among them is the Q-CHAT, a tool that has been translated, adapted, and validated in Chile by our team demonstrating evidence of Validity, Reliability and Sensitivity/Specificity suitable for use as population Screening (24, 25).

The Quantitative CHecklist for Autism in Toddlers, Q-CHAT (Allison et al., 2008) is an autism screening tool that conceptualizes the autism spectrum on a continuous scale, taking a dimensional approach to the identification of autistic traits (26–29). From a theoretical point of view, this proposal is consistent with the conceptual evolution of autism and with the quantitative nature of autistic traits as a continuum of symptoms and traits within the autism spectrum (30). The ability to dimensionally measure autistic traits using the Q-CHAT is supported by evidence showing that test scores are typically distributed across diverse populations studied (24, 26–28, 31, 32). In our study of adaptation and validation of the Q-CHAT in Chile (24), by means of an exploratory factor analysis we found a factor structure of 2 factors: a factor that groups the Socio-Communicative symptoms (Factor 1, “Communication and Social Interaction”) and a Factor that groups the repetitive behaviors (Factor 2, “Restrictive and Repetitive Patterns”). This version of the Q-CHAT excluded by rational test analysis item 18, leaving a 24-item version, the Q-CHAT-24. In a recent report (33), cut-off scores for the Q-CHAT-24 were established according to the harmonized optimal levels of Sensitivity/Specificity and the Youden Index. The cut-off point was 38, with a Sensitivity of 0.93, Specificity of 0.81 and Youden index of 0.70.

The overall objective of this study was to examine some psychometric characteristics of the Chilean-adapted version of the Quantitative Checklist for Autism in Toddlers (Q-CHAT-24) (24) in a group of unselected children (community sample). This version was administered remotely through an online version during the pandemic period to caregivers of children, aged 18–24 months, registered in four primary care polyclinics of the Health Service Araucanía Sur, Chile.

## 2 Materials and methods

### 2.1 Participants

A descriptive correlational design was used for this study. The sampling technique was non-probabilistic intentional. All primary caregivers of children aged 18–24 months who attended or were contacted remotely (by video call) for developmental follow-up during the quarantines periods in which this study was conducted (October 2020 to September 2021), in four primary care clinics in the Araucanía region of Chile during the study period (n=854), were invited to participate. Three hundred and seven (36.6%) caregivers agreed to participate.

The inclusion criteria were a) aged between 18 and 24 months at the time of the assessment, b) attended face-to-face with the primary caregivers or were contacted remotely for routine developmental follow-up checks. Primary caregiver was defined as the adult(s) responsible for the daily care of the young child (34). Exclusion criteria were a) not being accompanied by the primary caregiver(s), and b) presenting a genetic or neurological condition incompatible with the identification of the behaviors assessed (for example: severe gait disturbance, cerebral palsy, among others).

### 2.2 Instruments

#### 2.2.1 The Quantitative CHecklist for Autism in Toddlers Chilean version

In its original version (26) the Q-CHAT is a 25-item, parent- or caregiver-reporting scale designed as an autism screening instrument for children aged 18–24 months. The items are scored on a 5-point Likert-type scale (0–4), where higher scores indicate greater autistic traits. Each item is accompanied by a color illustration, which seeks to increase comprehensibility. Total Q-CHAT score >38 was established as the autism risk score.

For this research we used a culturally adapted version validated for Chile by our team (24). In this study we established evidence of concurrent validity with the M-CHAT-R/F, evidence of validity through adequate internal consistency (α=0.86) and established a 2-factor factor structure, excluding item 18 of the original Q-CHAT from the factor solution. A recent report (Gatica-Bahamonde, under review) established the optimal levels of Sensitivity/Specificity and Youden index (0.93/0.82; 0.76) for the 24-item Q-CHAT (Q-CHAT-24) using the cut-off point of 38 for a sample of Chilean preschoolers. This version was reproduced in its entirety and made available to researchers for subsequent dissemination through the REDCap online platform (35).

#### 2.2.2 The autism diagnostic observatory, second version

This is a standardized, semi-structured assessment of communication, social interaction, interests, and imaginative play, which defines the level of concern in relation to a possible diagnosis of ASD. This instrument consists of a set of precise activities, in a standardized context, in which the examinator observes behaviors relevant to the diagnosis of ASD. In this study, we used the Module T which is designed for children under 30 months of age.

#### 2.2.3 Socio-demographic questionnaire

A questionnaire constructed for this study was used, which seeks to collect sociodemographic data about the caregiver and his/her family, such as age, gender, marital status, years of schooling and socioeconomic level, among others.

### 2.3 Procedure

Between October 2020 and September 2021, all primary caregivers of children aged 18–24 months were invited to participate when they personally accompanied their children to developmental check-ups or when they were contacted remotely by phone or video call (depending on pandemic health conditions). Along with explaining the scope of the study, a link was sent to their mobile phones with access to the digital informed consent and to the REDCap platform where they could answer the Q-CHAT-24 and the socio-demographic questionnaire. When a caregiver agreed to answer the Q-CHAT-24, the results and the final score of their child were obtained immediately, together with indications regarding the steps to follow in case of obtaining a score of 37 or less (no suspected autism), they were instructed to follow their usual developmental controls. For scores of 38 or more (suspected autism), the indication was to wait to be contacted by the research team to start the study to confirm or exclude autism by medical assessment and the administration of ADOS-2 Module T. The study of suspected autism cases was conducted within three months of the Q-CHAT-24.

### 2.4 Ethical considerations

By signing (digitally) an informed consent, the free and voluntary participation, the confidentiality of data provided by the participants and the fact that they would be used only for research purposes were assured. This research was approved by the ethics committee of Araucanía Health Service, Chile.

### 2.5 Statistical analysis

The distribution of the total Q-CHAT scores was explored using the Shapiro Wilk (S-W) test. The results were contrasted with the distribution histogram and the standardized normal distribution probability plot (Q-Q Plot). As part of a rational item analysis, item-total correlations were examined using Pearson’s *r* parametric analyses. Reliability was estimated by examining internal consistency using Cronbach’s alpha for the total scale scores and separately for each of the factors previously found (24). As a measure of predictive validity, receiver operating characteristic (ROC) curves and area under the curve (AUC) with sensitivity, specificity, positive predictive value (PPV) and negative predictive value (NPV) were calculated for the cut-off points. Optimal cut-off points were selected based on their utility for screening purposes, following the proposal of Stevanovic et al. (29, 36): sensitivity and specificity > 0.8, sensitivity ≥ specificity, and Youden´s index ≥ 0.70.

## 3 Results

### 3.1 Socio-demographic characteristics

The mean age of the children evaluated was 21.16 months (median=20, range 18–24, SD=4.03), where 54.0% (n=169) were males. 78.2% (n=245) lived in urban sectors and 93.0% of the children belonged to families that had incomes below the 75th percentile of Chilean households, equivalent to less than US$1,140 (37). The mean age of caregivers was 30.0 years (median=30, range 18–60, SD=6.8). Of the caregivers who responded to the Q-CHAT, 93.5% (n=287) were mothers, 6.2% (n=19) were fathers, and 0.3% (n=1) were other caregivers. 78.8% (n=241) of the caregivers had 12 or more years of schooling.

**Table 1** summarizes the socio-demographic characteristics of the sample.

**Table 1:** Socio-demographic characteristics of the sample studied (n=307).

	n	%
Gender		
Male	169	54.0
Female	144	46.0
Go to the nursery	121	38.8
Family History		
Epilepsy	37	12.1
Language Disorder	48	15.5
Learning Disorder	41	13
Intellectual Disability	23	7.2
Psychiatric Disorder	38	12.3
Autism Spectrum Disorder	15	5.0
Location of the house		
Urban	245	78.2
Rural	68	21,8
Caregiver’s Education Level		
Basic Incomplete	15	4.9
Basic Complete	16	5.2
Secondary Incomplete	37	12.1
Secondary Complete	142	46.4
Superior Technical	44	14.4
Superior University	52	17.0
Employment status		
Employed	178	56.9
Unemployed	135	43.1
Family income		
Less than $USD190	28	9.1
$US 190 - $US 380	113	36.1
$US 380 - $US 630	100	32.0
$US 630 - $US 1,140	49	15.8
$US 1,140 - $US 1,500	11	3.4
Over $US 1,500	12	3.6

### 3.2 Q-CHAT-24 total scores distribution, item score distribution and item analysis

**Figure 1:**
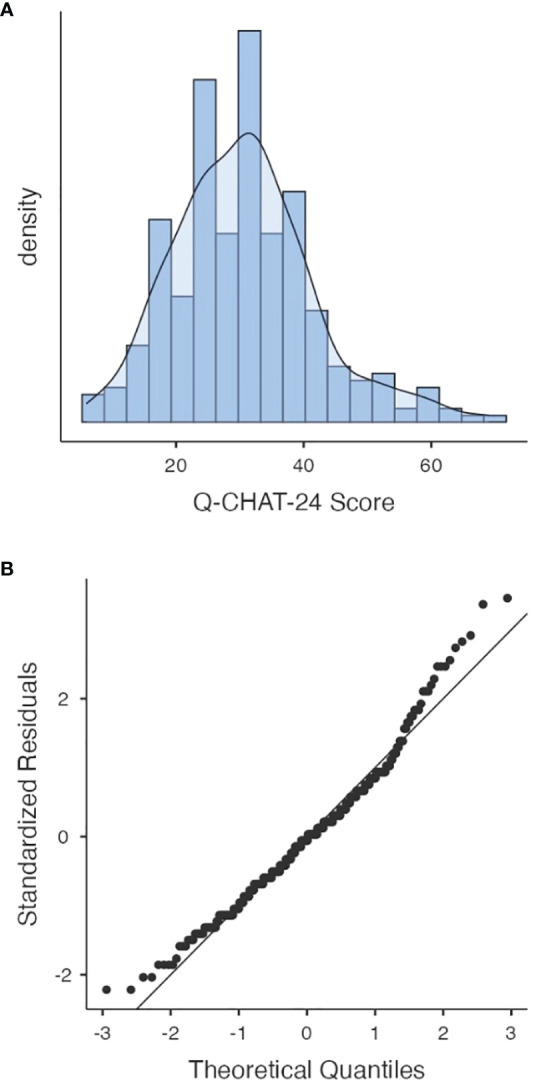
Histogram and Q-Q Plot for Q-CHAT-24 scores distribution in the studied sample (n=307). Figure shows distribution **(A)** and standardized normal probability plot **(B)** of the Q−CHAT total score in the screened population (n = 307).

For the Q-CHAT-24 the mean score for the whole sample analyzed was 30.62 (range 6–69, SD=11.1). The Shapiro-Wilk test revealed that the scores were normally distributed (S-W (307) =0.98, p<0.001) (**Figure 1**).

When analyzing scores by gender, the male Q-CHAT-24 mean score of 31.6 (SD=10.81, range 8–59) was higher than the female mean score of 27.98 (SD=9.71, range 6–63). These differences were statistically significant between the two groups (t(305) =2.44, p=0.01).

As part of a rational analysis of the Q-CHAT-24 items, an Item-Total correlation analysis (correlation between the Score of each item and the Total Score without considering the item to be analyzed) was performed. Each of the Q-CHAT-24 items was positively correlated with the Q-CHAT-24 Total Score. The item-total correlation was satisfactory (0.5>r>0.2) for most items, except for item 3 (r<0.2). The item-total analysis is shown in **Table 2**.

**Table 2:** Rational analysis of the items: Item-total Q-CHAT-24 Correlation (n=307).

Items	Item-total correlation
	(Pearson`s *r*)
1. Looks when called by name	0.59^‡^
2. Eye contact	0.58^‡^
3. Lines objects up^†^	0.13
4. Understands child’s speech	0.31^‡^
5. Protoimperative pointing	0.48^‡^
6. Protodeclarative pointing	0.54^‡^
7. Interest maintained by spinning object^†^	0.36^‡^
8. Number of words^†^	0.44^‡^
9. Pretend play	0.49^‡^
10. Follow a look	0.54^‡^
11. Sniff/lick unusual objects^†^	0.43^‡^
12. Use of hand as tool^†^	0.21^‡^
13. Walk on tiptoes^†^	0.28^‡^
14. Adapt to change in routine	0.32^‡^
15. Offer comfort	0.49^‡^
16. Do same thing over and over again^†^	0.41^‡^
17. Typicality of first words	0.48^‡^
19. Gestures	0.57^‡^
20. Unusual finger movements^†^	0.45^‡^
21. Check reaction	0.22^‡^
22. Maintenance of interest^†^	0.33^‡^
23. Twiddle objects repetitively^†^	0.45^‡^
24. Oversensitive to noise^†^	0.38^‡^
25. Stare at nothing with no purpose^†^	0.54^‡^

^†^Reverse-scored items.

^‡^Satisfactory correlation (0.5≥r≥0.2).

### 3.3 Q-CHAT-24 reliability

Internal consistency was acceptable for the Q-CHAT-24 (Cronbach ´s α=0.78). The internal consistencies were analyzed for the Q-CHAT-24 Factors, and they were good for factor 1 “Communication and Social Interaction” (Cronbach ´s α=0.85) and acceptable for factor 2 “Restrictive and Repetitive Patterns” (Cronbach ´s α=0.74).

### 3.4 Comparison between Q-CHAT-24 and ADOS-2 module T scores

Seventy-one cases (23.12%) scored 38 points or more on the Q-CHAT-24, qualifying as Autistic Risk. The mean score in the Autism Risk group was 46.9 (range 38–69, SD=8.0) and the mean score in the Non-Autism Risk group was 26.09 (range 6–37, SD=7.23). The differences observed were statistically significant (t(305) =-19.54, p<0.001).

**Figure 2:**
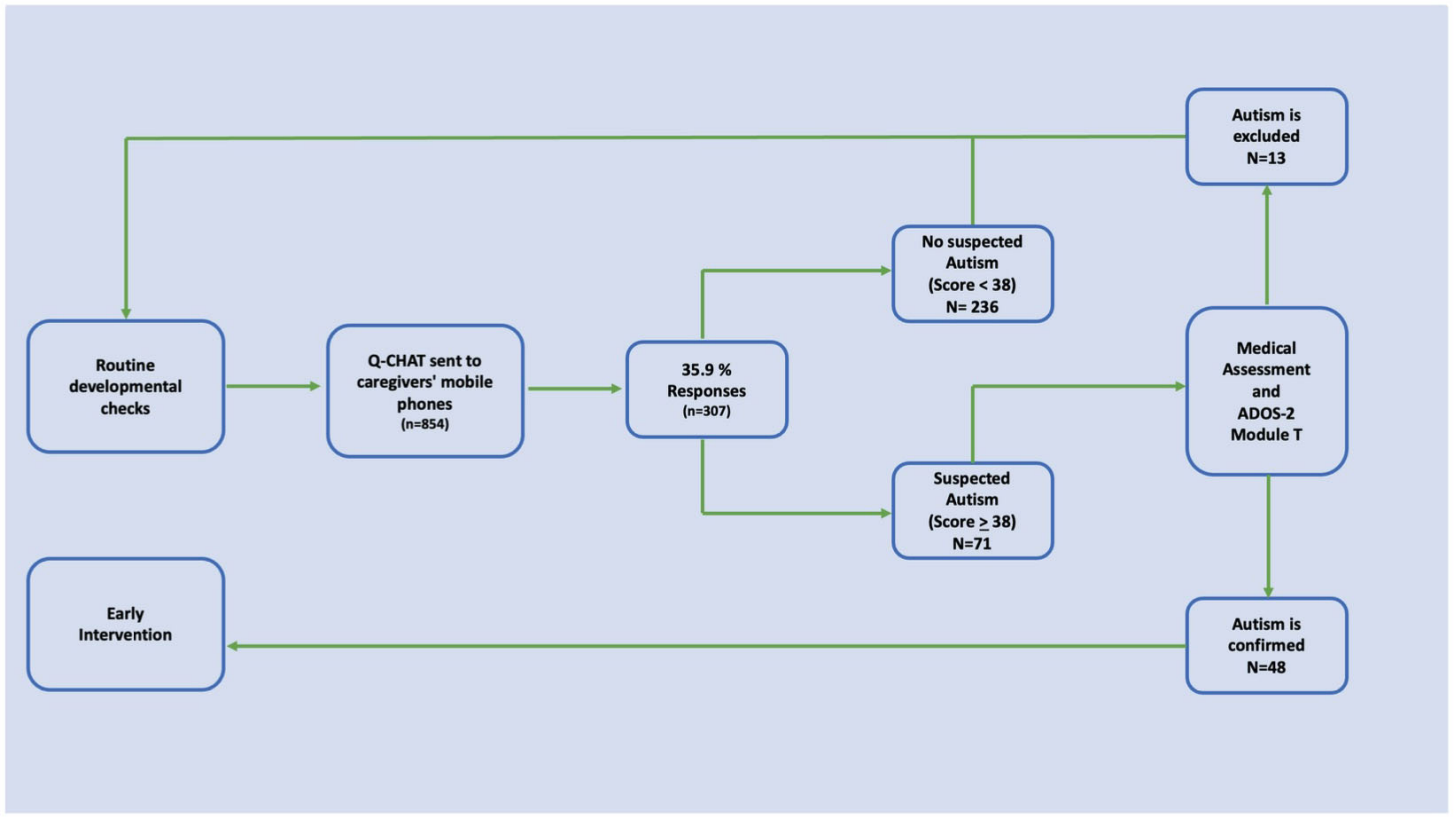
Study design overview and selection of participants (n=307). Eight hundred and fifty-four caregivers were invited to participate. Of these, 307 agreed to participate by signing the informed consent form and completed the socio-demographic questionnaire and the Q-CHAT-24. Seventy-one children (23.1%) were identified as being at risk for autism. Of these, 48 were confirmed by the ADOS-2 module T as Autism.

Of the cases identified as Autism risk, 10 caregivers of children at risk for autism did not agree to undergo with the ADOS-2 confirmatory assessment. 48 cases (78.6%) were confirmed as Autism by the ADOS-2 Module T and 13 (21.3%) were excluded as such. As a control measure, 22 non-risk children were assessed with the ADOS-2 Module T, and all of them were excluded as autistic. **Figure 2** summarizes the study design and the selection of participants according to their classification as Autism Risk/Non-Risk according to the Q-CHAT-24 and according to the Autism Confirmed/Excluded status according to the ADOS-2 Module T.

The mean score of the Q-CHAT-24 in the group of cases Autism confirmed was 48.23 (range 38–69, *SD*=8.44) and 37.17 (range 28–44, *SD*=3.19) for the group of Autism excluded. The observed differences were statistically significant (*M-W* =122, p<0.001).

**Figure 3:**
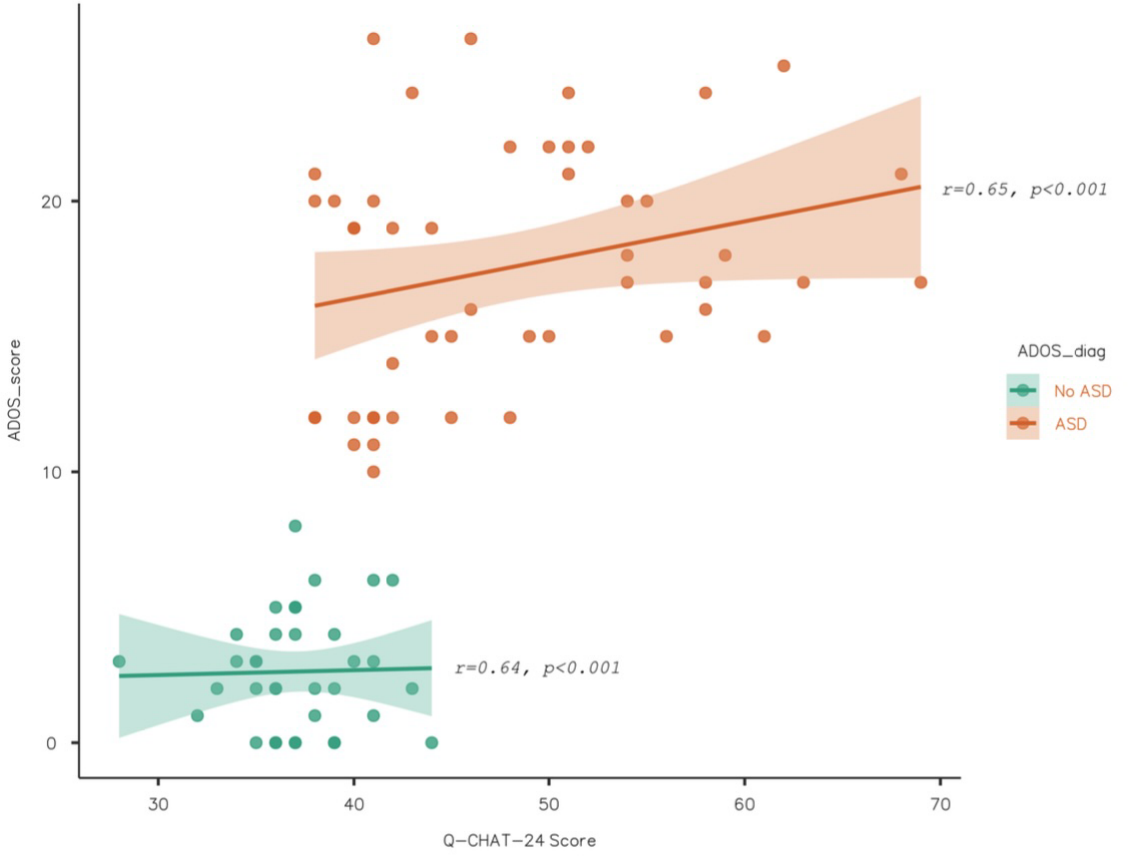
ADOS-2 and Q-CHAT-24 Scatterplot (n=307). Figure shows a Scatterplot with a tendency curve to graph the correlation between ADOS-2 Module T and Q-CHAT-24 scores in both ASD and No-ASD groups. The correlation between these scores was high, positive, and statistically significant in both groups (r=0.65, p<0.001 and r=0.65, p<0.00, respectively).

The correlation between scores on the Q-CHAT-24 and scores on the ADOS-2 Module T was positive and statistically significant (*r*=0.65, *p*<0.001). This correlation was high, positive and significant for the Confirmed and Excluded Autism groups (*r*=0.65 and *r*=0.64, respectively). **Figure 3** shows a scatter plot with the trend curve for the correlation between scores on the ADOS-2 Module T and the Q-CHAT-24 in the ASD and non-ASD groups.

### 3.5 Predictive validity Q-CHAT-24

**Figure 4:**
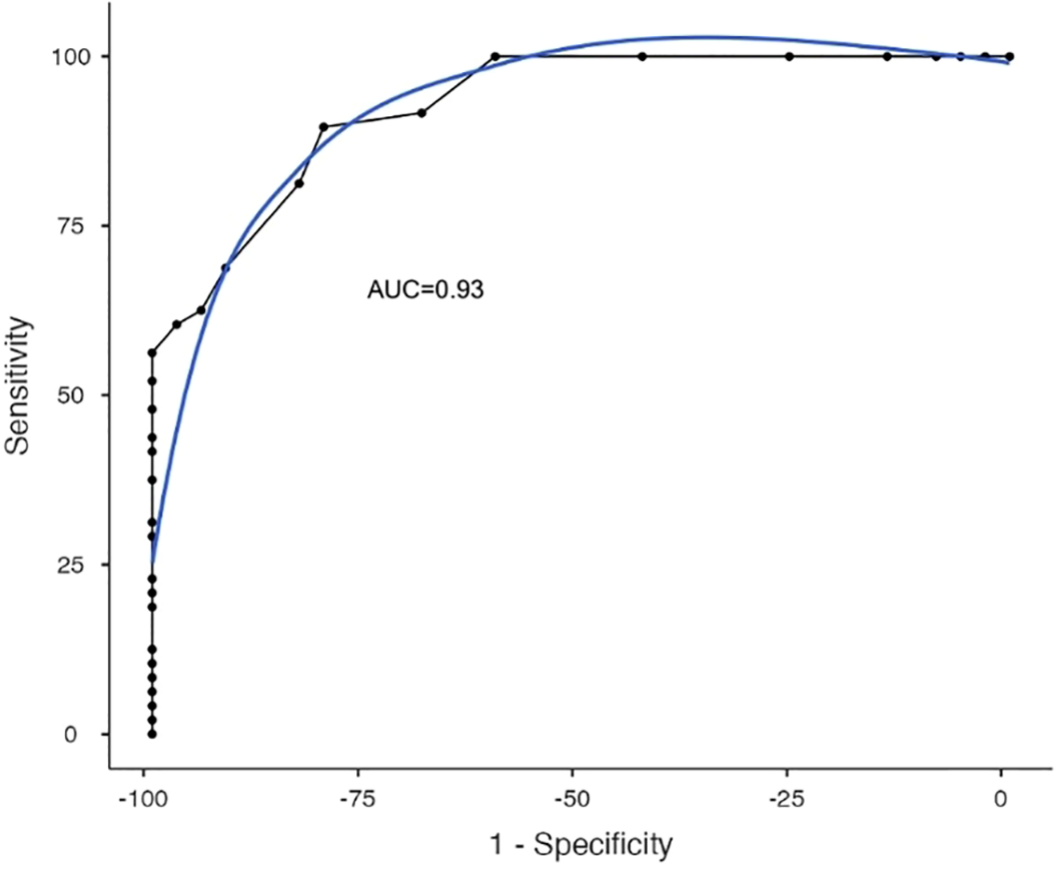
Q-CHAT-24 ROC Curve. The figure shows the receiver operating characteristic (ROC) curve analysis of Q-CHAT-24 in the studied sample. The AUC values were 0.93 with statistical significance (p<0.01).

Receiver operating characteristic (ROC) curve analyses were performed. The AUC values were 0.93 with statistical significance (p<0.01). For the cut-off point of 38, the Sensitivity, Specificity and Youden index values were 0.89, 0.8 and 0.7, respectively. The Positive Predictive Value (PPV) was 86% and the Negative Predictive Value (NPV) was 85%. **Figure 4** shows the ROC curve of the described analysis.

## 4 Discussion

The aim of this study was to examine some psychometric characteristics of the Chilean-adapted version of the Quantitative Checklist for Autism in Toddlers (Q-CHAT-24) (14) in a group of unselected children (community sample). This version was administered remotely through an online version during the pandemic period to caregivers of children, aged 18–24 months, registered in four primary care polyclinics of the Health Service Araucanía Sur, Chile.

Q-CHAT-24 total score showed a normal distribution, replicating previous findings and in other cultural contexts, both in community sample (24, 26–29) and confirms the quantitative and dimensional nature with which Q-CHAT was conceived (26).

We found significantly higher scores for boys than for girls. These results are consistent with epidemiological and genetic evidence showing that males are associated with higher autistic traits in general population samples (38–40).

The high percentage of children scoring above the cut-off point is consistent with previous findings from the use of the Q-CHAT in the Chilean population reported by Roman-Urrestarazu et al. (24). This percentage is probably determined by the fact that the Q-CHAT-24 identifies autism risk in a heterogeneous child population that shares manifestations with other developmental trajectories such as neurodevelopmental delays.

Rational analysis of the Q-CHAT items showed that 23 of the 24 items have item-total correlations with satisfactory values, which is quantitatively higher than that observed in the 25-item version in the Chilean population (24).

Reliability analysis show acceptable values for the overall scale (α=0.78) and for factor 2”Communication and Social Interaction” (α=0.74), and good for factor 1 “Restrictive and Repetitive Patterns” (α=0.85).

Finally, regarding the evidence of predictive validity, the Q-CHAT-24 showed Sensitivity and Specificity values that met the criteria defined *a priori* as necessary to be used for screening: sensitivity and specificity > 0.8, sensitivity ≥ specificity, and Youden index ≥ 0.70. Interestingly, for Q-CHAT-24 the cut-off point that best harmonizes the optimal values of sensitivity and specificity (cut-off point of 38), is higher than the one originally proposed by Allison et al. (2008) (26) and replicated by Stevanovic (2021) (29), for a longer version (25 items). As De Leeuw et al. (2020) suggest (41), it is possible that the higher quantification of symptoms observed in our study in relation to other reports is due to the cultural differences that can be observed between cultures in the quantification of autism symptoms. For example, Magaña and Smith (2013) (42) reported lower parental concern in parents of Latino children compared to parents of White American children.

Considering the polytomous nature of the Q-CHAT-24 items, sensitivity and specificity values probably do not account for the full complexity of the test and should be complemented with Predictive Value metrics. In our study, the observed PPV value is high (86%) but lower than those observed by Roman-Urrestarazu et al. (2021) (25)in another sample of Chilean population.

### 4.1 Limitations

The low response rate (36.6%) is an important limitation of this study and is likely related to self-selection bias and the high rate of scores above the cut-off point for autism risk (23.12%) observed in our sample. This may limit the generalizability of these results to a general population sample.

The study design did not consider the application of the diagnostic Gold Standard (ADOS-2 Module T) (43) to an equal number of control cases without risk of autism, so the specificity values of the test should be taken with caution.

### 4.2 Conclusions

In accordance with the objectives of this study, evidence of reliability and predictive validity was demonstrated for the Q-CHAT-24 in this Chilean population. More importantly, this study provides Sensitivity and Specificity data for a remote application version of an autism screening tool already validated in Chile. The implications of this have to do with the possibility of establishing a remote assessment system for children at risk of autism on a population scale.

Future directions following this study include examining the Q-CHAT-24 as a measure of early detection of autism in larger samples and conducting other follow-up studies and thus confirming the evidence of predictive validity. Similarly, it would be useful to incorporate other developmental variables such as measures of language and social development in future studies.

Finally, prospective studies could provide evidence on the eventual impact that isolation and lack of socialization may have had on Autism diagnosis rates during the period of the COVID 19 pandemic.

## Data availability statement

The datasets presented in this article are not readily available because the data sets generated and analyzed during this study are not publicly available because of our agreement with the parents of the children. Requests to access the datasets should be directed to gabriel.gatica@uc.cl.

## Ethics statement

The studies involving humans were approved by Ethics Committee of Araucanía Health Service, Chile. The studies were conducted in accordance with the local legislation and institutional requirements. Written informed consent for participation in this study was provided by the participants’ legal guardians/next of kin.

## Author contributions

GG-B: Writing – review & editing, Writing – original draft, Visualization, Validation, Supervision, Software, Resources, Project administration, Methodology, Investigation, Funding acquisition, Formal analysis, Data curation, Conceptualization. AM-F: Writing – review & editing, Writing – original draft, Validation, Supervision, Formal analysis, Data curation. FS-S: Writing – review & editing, Writing – original draft, Resources, Investigation. CP-D: Writing – review & editing, Writing – original draft, Resources, Investigation. RK: Writing – review & editing, Writing – original draft, Methodology, Investigation, Formal analysis, Data curation. KC: Writing – review & editing, Writing – original draft, Supervision, Methodology, Investigation, Formal analysis, Conceptualization. AR-U: Writing – review & editing, Writing – original draft, Supervision, Methodology, Investigation, Formal analysis, Conceptualization.
